# Seaweed Extracts Improve Salinity Tolerance in Cereal Crops—A Meta‐Analysis

**DOI:** 10.1002/pei3.70094

**Published:** 2025-10-28

**Authors:** Md. Nuruzzaman, Md. Tahjib‐Ul‐Arif, Md. Abdul Hannan, Yoshiyuki Murata, M. Afzal Hossain

**Affiliations:** ^1^ Department of Plant Resources College of Industrial Sciences, Kongju National University Yesan Republic of Korea; ^2^ Department of Biochemistry and Molecular Biology Bangladesh Agricultural University Mymensingh Bangladesh; ^3^ Graduate School of Environmental, Life, Natural Science and Technology Okayama University Okayama Japan

**Keywords:** abiotic stress, crop tolerance, marine algae, plant growth, salt stress, sustainable agriculture

## Abstract

Seaweeds are considered an essential component of the blue economy. Because seaweed extracts are rich in bioactive compounds that enhance plant stress resilience, exploiting this resource could offer a sustainable solution for crop production. Salinity is a major abiotic challenge that significantly impacts crop yield and food security. Through meta‐analysis, we explored whether the exogenous application of seaweed extracts improves the salt tolerance of cereal crops. All the studies chosen for this study utilized aqueous seaweed extracts as foliar sprays. A multi‐level meta‐analysis with a mixed effects model was performed to determine the effect size. This meta‐analysis demonstrated that applying aqueous seaweed extracts enhanced the shoot and root biomass under normal and salinity stress conditions, suggesting that seaweed extract can help improve crop stress tolerance. The seaweeds studied belonged to three classes: *Phaeophyceae*, *Rhodophyta*, and *Chlorophyta*, with extracts from *Chlorophyta* and *Phaeophyceae* significantly enhancing biomass production under salinity conditions. Applying aqueous seaweed extracts effectively improved salinity tolerance at both 34.2–100 mM and 101–400 mM NaCl equivalent salinity stress. Moreover, exogenous foliar application of ≤ 25% aqueous seaweed extracts was most effective for improving salinity tolerance in cereals. The impact of seaweed extracts on cereal crop yields has not been extensively reported; therefore, further studies should focus on this aspect.

## Graphical abstract

Meta‐analysis demonstrated that applying aqueous seaweed extract enhanced the shoot and root biomass under salinity stress conditions. Applying aqueous seaweed extract (0.2%–10%) effectively improved salinity tolerance, both 34.2–100 mM NaCl and 101–400 mM NaCl equivalent salinity stress.

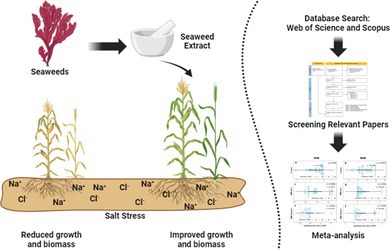

## 1 Introduction

Sustainable agriculture is critically important because it supplies essential raw materials for food security and hunger eradication, supports sustainable livelihoods, and consistently contributes to global economic stability (Nhemachena et al. 2020; Mondal et al. 2022). The world population is anticipated to reach nearly 10 billion by 2050 (Duro et al. 2020). To meet the demand, the agri‐food industry faces the severe challenge of increasing food production by 60%–110% (Rockström et al. 2017; Ben Ayed and Hanana 2021). The shortage of natural resources and rapid industrialization, along with increasing greenhouse gas emissions, hinder agricultural extension in terms of both quality and quantity (Mondal et al. 2022). Moreover, due to prominent changes in climate patterns, climate extremes like droughts, sea‐level rise, flooding, and salinity dramatically influence agricultural areas (Corwin 2021; Roy et al. 2023).

Salinity is a real danger to agriculture that promptly restricts the sustainability of agriculture and the economy by reducing the productivity and suitability of the land area for most field crops (Butcher et al. 2016). Further, the level of soil salinization worldwide is still increasing. It is estimated that about 20% of cropped fields and 33% of irrigation fields are affected by salinity (Mukhopadhyay et al. 2021), and by 2050, over half of arable land will be susceptible to salinization (Tahjib‐Ul‐Arif et al. 2018). A shift from the present focus on boosting productivity toward prioritizing agricultural sustainability is urgently required. Scientists have developed physical, chemical, and biological methods to resolve the salinity stress and enhance crop yields. However, the expense, lack of an intense soil conditioner, and good irrigation water frequently limit the implementation of these methods. In response, cost‐effective and environmentally friendly strategies such as biocompatible soil amendments or foliar applications have been introduced, where algal or seaweed extracts are applied as bio‐stimulants to promote plant growth and increase salinity tolerance (Mukherjee and Patel 2020; Mukhopadhyay et al. 2021).

Seaweed includes multicellular and microscopic marine algae from several taxonomic groups, including brown seaweed (around 2000 species under *Phaeophyceae*), red seaweed (over 7200 species under *Rhodophyta*), and green seaweed (over 1800 macroalgae species under *Chlorophyta*). These marine organisms are well known for their richness in growth‐promoting constituents and bioactive compounds such as phytohormones (auxins, gibberellic acid, cytokinins, etc.), inorganic elements (Ca, Mg, K, etc.), polysaccharides, amino acids, lipids, proteins, phenolics, and vitamins (Cai et al. 2021; Sapatinha et al. 2022). Seaweeds such as *Ulva rigida* can be applied as foliar treatments, and their biological effects are revealed by improving plant growth, yield, and product quality and increasing tolerance to abiotic stress like salinity (Ali et al. 2021; Latique et al. 2021). Seed priming with *Sargassum angustifolium* and *Spirulina platensis* extract alleviates the negative effect of salt stress on milkweed plants (Bahmani Jafarlou et al. 2021). These treatments enhance seed germination, biomass allocation, shoot and root length, and root/shoot ratios, consequently improving salt tolerance. Besides, a pot experiment with *Vigna sinensis* and maize ( *Zea mays* ) showed that seed priming with extract from *Laurencia obtusa* , *Ulva fasciata* , and *Cystoseira compressa* at 20 g/L promotes seed germination and seedling growth of both species and alleviates the negative drawbacks of salinity stress (Hussein et al. 2021). Polysaccharides from *Pyropia yezoensis* and an acid heteropolysaccharide from *Macrocystis pyrifera* enhance the salt‐stress tolerance of wheat plants (Zou et al. 2018, 2021). Studies revealed that *Halimeda opuntia* and *Padina pavonica* extracts enhance maize growth and metabolic activity under saline soil conditions (Attia et al. 2022). Additionally, applying extracts from *Hormophysa cuneiformis* and *Actinotrichia fragilis* to wheat seeds in saline environments increased growth performance and enhanced the tolerance of salt stress by improving photosynthetic pigments, phenolic compounds, osmolytes, nutrient uptake, as well as activating antioxidant enzymes (Abdel Latef et al. 2021). Likewise, algal amendments have been shown to improve grain yield and grain quality of rice by alleviating the impacts of salt and water stress (El‐Katony et al. 2021).

Seaweed extracts offer a natural and sustainable means to enhance plant salt tolerance, especially at low concentrations, and have long been used by coastal farmers as organic fertilizers (Singh et al. 2025). Their biodegradability and compatibility with existing agricultural systems make them eco‐friendly alternatives to synthetic inputs (Sekar et al. 2025). To assess their effectiveness against salinity, we systematically review the available studies in cereal crops (rice, wheat, and maize) through a meta‐analysis approach. Our goal is to unveil whether seaweed extracts can alleviate salinity stress in cereal crops. Specifically, we aim to answer:

- Do seaweed extracts improve cereal crop growth under non‐stress and salinity stress conditions?
- Do seaweed extracts from different seaweed species affect the growth of cereal crops differently under salinity stress?
- Do seaweed extracts have similar effects on various cereal crops?
- What concentration of seaweed extract is most effective in improving salinity tolerance?
- Up to what level of salinity can seaweed extracts improve plant growth?

## 2 Materials and Methods

### 2.1 Literature Search and Data Collection

**FIGURE 1:**
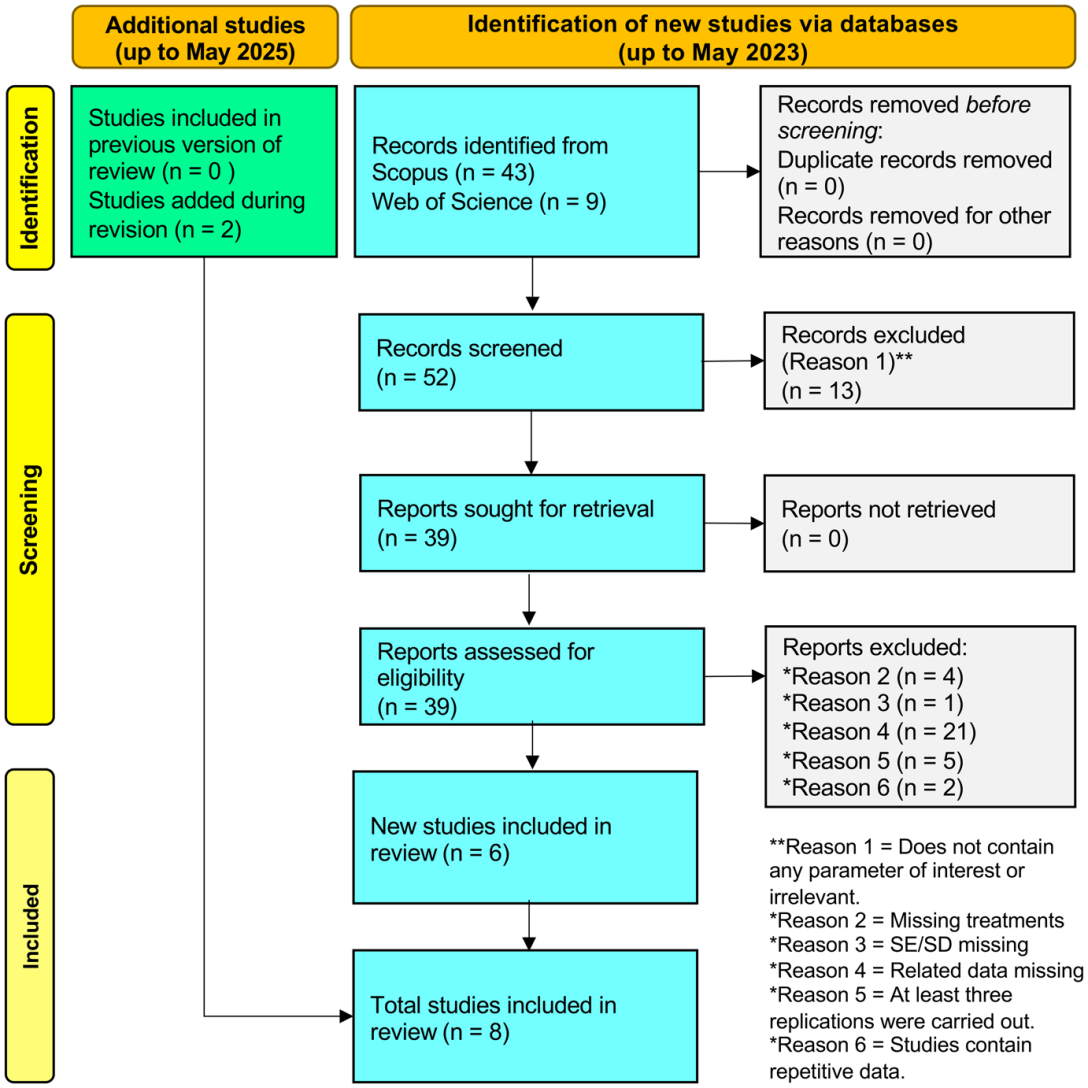
PRISMA flow diagram showing the systematic search and screening process used in this meta‐analysis.

We searched for research articles in May 2023 using the Web of Science (in May 2025 using Scopus) (http://webofknowledge.com/) and Scopus (https://www.scopus.com/). We followed the general procedures to acquire data for the meta‐analysis (Field and Gillett 2010). The following combinations of search terms were used to build our database: (i) “seaweed*” or “alga*”, and (ii) “wheat” or “ *Triticum aestivum* ” or “maize” or “ *Zea mays* ” or “rice” or “ *Oryza sativa* ”, and (iii) “salinity” or “salt” or “NaCl” or “saline”. We also searched articles for other cereals, such as barley and oats, but did not find any relevant papers. Based on the title and abstract of the search results, 52 articles were identified as likely to contain relevant data (Figure 1). We downloaded the full texts of selected articles for further screening. Studies were selected if they met the following requirements: (i) plants were salt‐stressed and treated with foliar seaweed extract; (ii) experiments included wheat, maize, or rice; (iii) plants were grown in field, pot, or greenhouse conditions; (iv) growth parameters (e.g., shoot/root biomass) were reported with control data; and (v) studies provided at least three replicates with standard deviations, standard errors, or other relevant statistical information for effect size calculation. Moreover, studies involving transgenic or mutant plants were excluded. In May 2025, we conducted a systematic search to identify recently published articles and found two additional relevant studies. Ultimately, eight studies were selected for data extraction and meta‐analysis (Figure 1 and Table S1). The composition of seaweed extracts varies significantly depending on the extraction method. Notably, all studies included in our analysis used aqueous seaweed extracts.

### 2.2 Data Extraction

We extracted four key growth parameters from the selected studies: shoot fresh weight (SFW), shoot dry weight (SDW), root fresh weight (RFW), and root dry weight (RDW). Numerical data were obtained from figures using WebPlotDigitizer (https://automeris.io/WebPlotDigitizer/). For each parameter, we recorded the mean values, number of replications, and corresponding measures of variability (SD or SE). When only SE was reported, we converted it to SD using the formula: SD = SE × √*n*, where ‘*n*’ is the number of replications. Yield data were not included in the analysis, as only two studies reported this parameter.

### 2.3 Meta‐Analysis

All analyses and visualizations were performed with the R 4.4.1 program. A meta‐analysis approach was used to determine the effect of seaweed extracts on the growth of cereal crops under salinity stress. Effect sizes were calculated using the log response ratio (ROM) (Hedges et al. 1999), defined as the natural logarithm of the ratio of the mean outcome in the treatment group to the control group. Sampling variances for each effect size were computed using the *escalc()* function from the ‘metafor’ R package. The variance of each study was calculated based on means, number of replications, and standard deviation (SD) of different treatments (Hedges et al. 1999; Luo et al. 2006). The effect size and its variance for each comparison were saved for further analysis.

$$\mathrm{ROM}=\ln\left(\text{Treatment mean}/\text{Control mean}\right)$$

A multi‐level random‐effects meta‐analysis model was fitted using the *rma.mv()* function from ‘metafor’ (Viechtbauer 2010). Model parameters were calculated using the restricted maximum likelihood estimator (REML) (Viechtbauer 2005). Study site and effect size were considered random factors in the meta‐analysis as several study sites contributed more than one effect size. All plots are presented as orchard plots using the ‘orchaRd’ package in R (Nakagawa et al. 2021). Orchard plots, in addition to showing overall mean effects and confidence intervals from meta‐analyses, also include 95% prediction intervals. Confidence intervals represent the range of the average actual effect to be found, and prediction intervals show the range in which 95% of effects are anticipated to occur in future studies of a similar nature. Residual heterogeneity was assessed using the QE statistic (Table S2). Publication bias was assessed with Egger's regression tests (Sterne and Egger 2005) (Table S2).

### 2.4 Publication Bias Adjustment and Sensitivity Analyses

To assess publication bias and evaluate the robustness of the meta‐analytic results, we conducted a random‐effects meta‐analysis for continuous outcomes using the *metacont()* function from the ‘meta’ package in R. To further explore the potential impact of small‐study effects, we performed a limit meta‐analysis using the *limitmeta()* function from the ‘metasens’ package, based on the results from *metacont()*. This method re‐estimates the overall effect size under the assumption of no small‐study effects (Rucker et al. 2011), thereby providing an adjustment for potential publication bias. A funnel plot was generated from the *limitmeta()* output, displaying both adjusted and unadjusted effect sizes. The difference between these estimates was used to assess the robustness and sensitivity of the main findings. All analyses and visualizations were performed with the R 4.4.1 program.

## 3 Results

### 3.1 Effects of Seaweed Extract on Shoot Growth

**FIGURE 2:**
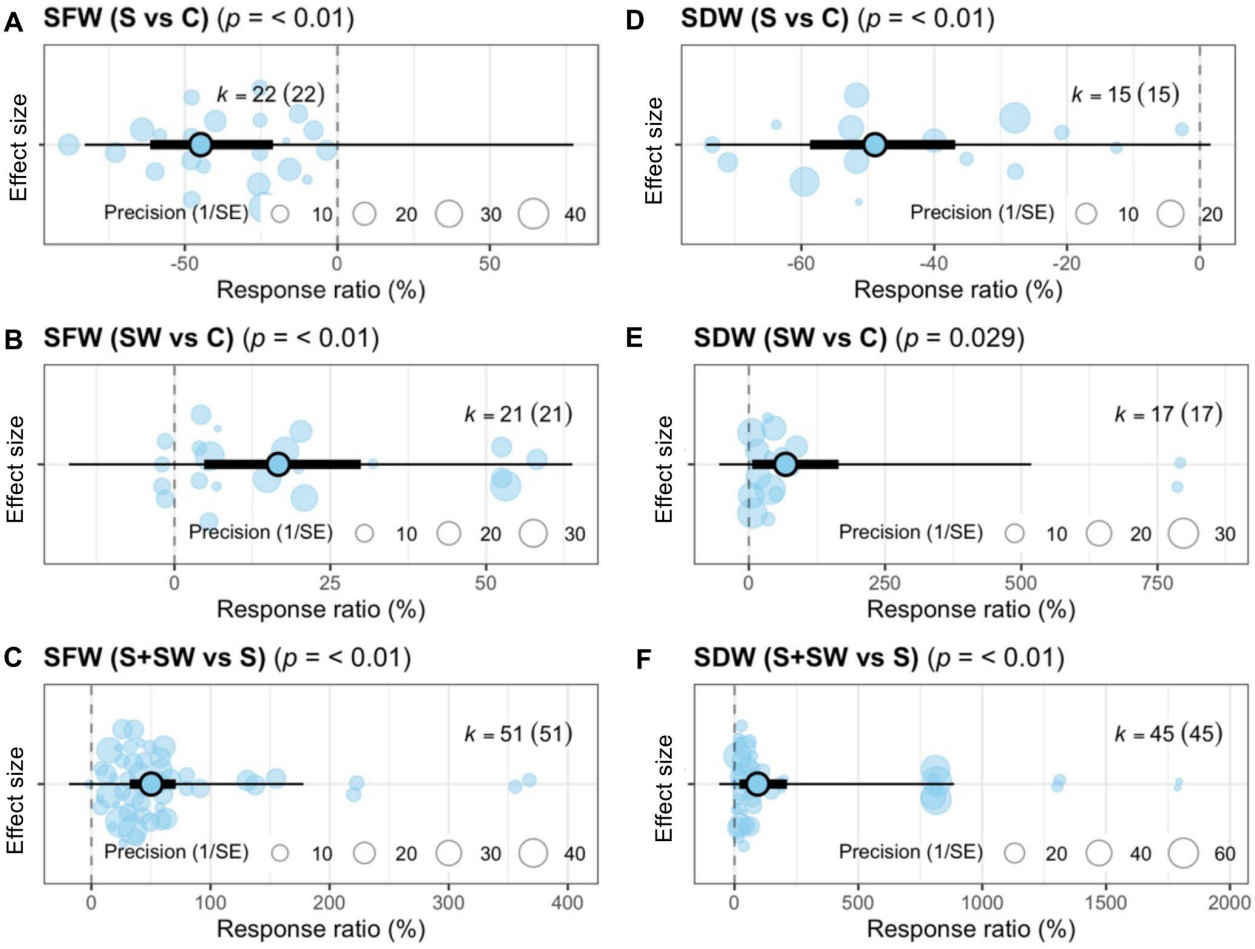
Effects of seaweed extract on shoot fresh weight (SFW) and shoot dry weight (SDW) of cereal crops (wheat, maize, and rice) grown under salinity conditions. Plots show means, 95% confidence interval (CI, thick whisker), 95% prediction interval (PI, thin whisker), and individual effect sizes scaled by their precision (circles). *k* indicates the number of studies included in this study, and *p* < 0.05 indicates the significant effect of a particular treatment. The dashed vertical line shows the null response ratio (%). C (control), S (salinity), SW (seaweed extract foliar spray), and S + SW (salinity + seaweed extract foliar spray) are different treatment combinations.

**FIGURE 3:**
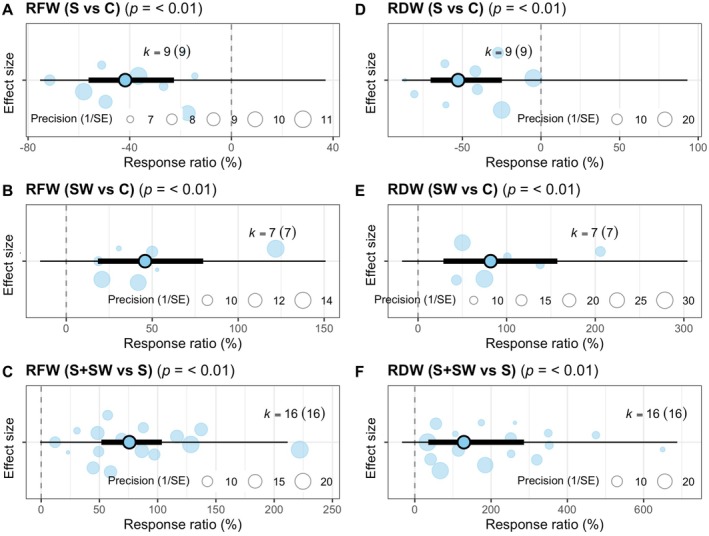
Effects of seaweed extract on root fresh weight (RFW) and root dry weight (RDW) of cereal crops (wheat, maize, and rice) grown under salinity conditions. Meta‐analytic plots show means, 95% confidence interval (CI, thick whisker), 95% prediction interval (PI, thin whisker), and individual effect sizes scaled by their precision (circles). *k* indicates the number of studies included in this study, and *p* < 0.05 indicates the significant effect of a particular treatment. The dashed vertical line shows the null response ratio (%). C (control), S (salinity), SW (seaweed extract foliar spray), and S + SW (salinity + seaweed extract foliar spray) are different treatment combinations.

We performed a meta‐analysis to assess the effect of seaweed under salinity stress conditions in cereal crops (rice, wheat, and maize). Then, we evaluated its impacts on the growth parameters of cereal crops, such as SFW, SDW, RFW, and RDW. All assessed growth parameters were significantly affected by seaweed extracts under salinity stress (Figures 2 and 3).

Compared to control, salinity (S) decreased both SFW and SDW significantly (*p* < 0.01 and *p* < 0.01, respectively) in cereal crops (Figure 2A,B). Only seaweed extract treatment exhibited a significantly positive effect on SFW (*p* < 0.01) but not a significant improvement in SDW (*p* < 0.01) (Figure 2C,D). However, compared to the salinity condition, the SW + S treatment increased both SFW and SDW significantly (*p* < 0.01 and *p* < 0.01, respectively) (Figure 2E,F).

### 3.2 Effects of Seaweed Extract on Root Growth

The effect trend of SW + S condition on root fresh weight (RFW) and root dry weight (RDW) of cereal crops under salinity stress was similar to those on the SFW and SDW. Results indicated that salinity stress reduced both RFW and RDW significantly (*p* < 0.01 and *p* < 0.01, respectively) compared to the control condition (Figure 3A,B). Seaweed increased RFW and RDW with *p*‐values < 0.01 and 0.01, respectively (Figure 3C,D). Nevertheless, SW + S treatment showed a significant increase in both RFW and RDW (*p* < 0.01 and *p* < 0.01, respectively) as compared to the saline condition (Figure 3E,F).

### 3.3 Effects of Different Types of Seaweed Extracts on Cereal Growth Under Salt Stress

**FIGURE 4:**
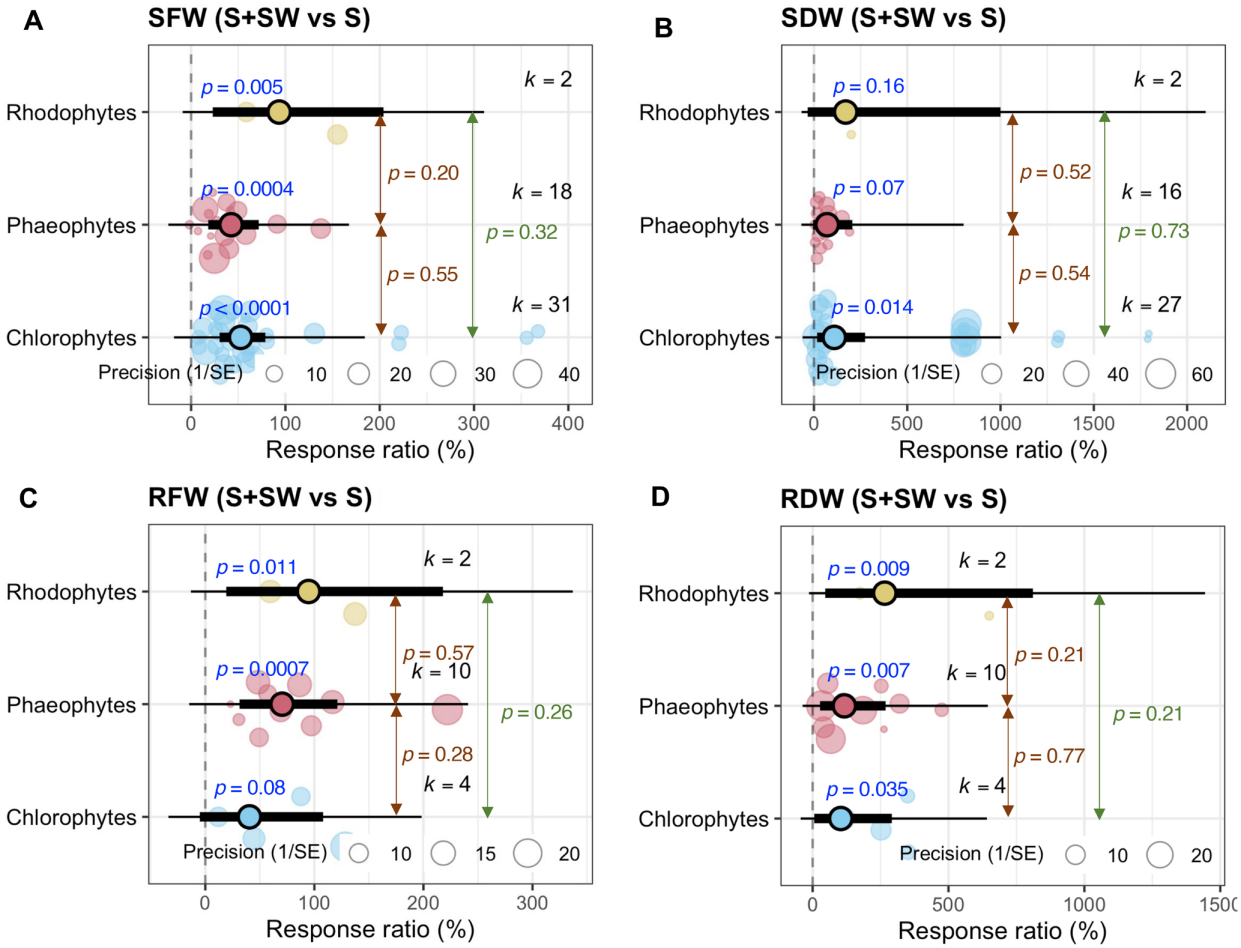
Effects of seaweed types on shoot fresh weight (SFW), shoot dry weight (SDW), root fresh weight (RFW), and root dry weight (RDW) of cereal crops (wheat and maize) grown under salinity conditions. Plots show means, 95% confidence interval (CI, thick whisker), 95% prediction interval (PI, thin whisker), and individual effect sizes scaled by their precision (circles). *k* indicates the number of studies included in this study, and *p* < 0.05 indicates the significant effect of a particular treatment. The dashed vertical line shows the null response ratio (%). S (salinity) and S + SW (salinity + seaweed extract foliar spray) are different treatment combinations.

Application of extracts from all three types of seaweed, rhodophytes, phaeophytes, and chlorophytes, to salt‐stressed cereals significantly improved the SFW (*p* = 0.005, *p* = 0.0004, and *p* < 0.0001, respectively) compared to salt‐stressed cereals without seaweed extract (Figure 4A). There was no significant difference in the extent of SFW improvement among the different types of seaweed extracts (Figure 4A). Among the extracts from rhodophytes, phaeophytes, and chlorophytes, only the chlorophyte extract application to salt‐stressed cereals significantly improved the SDW (*p* = 0.014) compared to salt‐stressed cereals without seaweed extract (Figure 4B). There was no significant difference in the extent of SDW improvement among the different types of seaweed extracts (Figure 4B). Salt‐stressed cereals treated with seaweed extracts from rhodophytes and phaeophytes showed a significant increase in RFW (*p* = 0.011 and *p* = 0.0007, respectively) and RDW (*p* = 0.009 and *p* = 0.007, respectively) compared to untreated salt‐stressed controls (Figure 4C,D). However, there were no significant differences in the extent of RFW and RDW improvement among the different types of seaweed extracts (Figure 4C,D). It is important to note that the number of studies evaluating the effects of different seaweed types was limited, particularly for rhodophytes in relation to SFW and SDW (*k* = 2), and for rhodophytes and chlorophytes in relation to RFW and RDW (*k* = 2 and *k* = 4, respectively), which may limit the confidence in drawing definitive conclusions about their effects on cereal crops.

### 3.4 Effects of Seaweed Extracts on Growth of Rice, Maize, and Wheat Under Salt Stress

**FIGURE 5:**
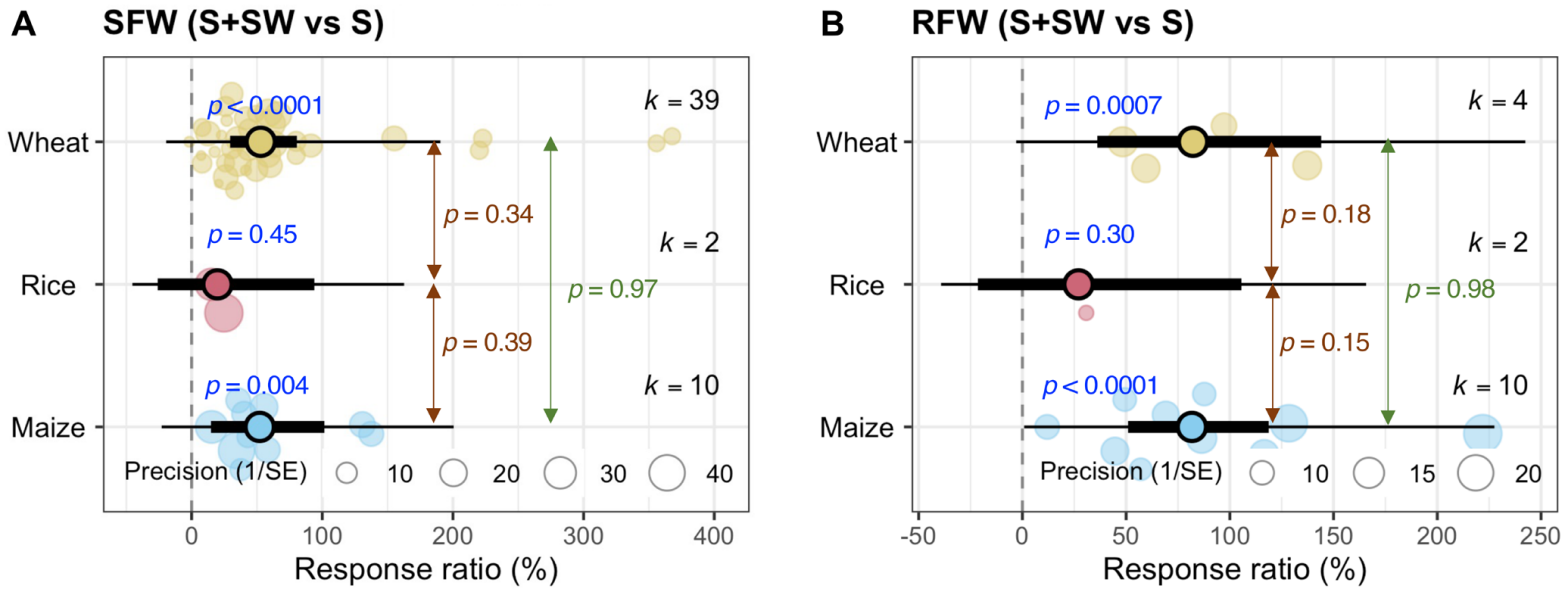
Seaweed extract‐mediated changes in shoot fresh weight (SFW) and root fresh weight (RFW) of rice, wheat, and maize under salinity conditions. Plots show means, 95% confidence interval (CI, thick whisker), 95% prediction interval (PI, thin whisker), and individual effect sizes scaled by their precision (circles). *k* indicates the number of studies included in this study, and *p* < 0.05 indicates the significant effect of a particular treatment. The dashed vertical line shows the null response ratio (%). S (salinity) and S + SW (salinity + seaweed extract foliar spray) are different treatment combinations.

The response ratio of SFW under the combined S + SW treatment compared to S alone for wheat, rice, and maize was presented in Figure 5A. Wheat exhibited a significant positive response (*p* < 0.0001, *k* = 39), indicating a strong and consistent increase in shoot biomass by seaweed extract. Maize also showed a significant increase (*p* = 0.004, *k* = 10). In contrast, rice did not show a significant response (*p* = 0.45, *k* = 2), likely due to the small number of observations. Pairwise comparisons between crop types showed no statistically significant differences (wheat vs. rice: *p* = 0.34; rice vs. maize: *p* = 0.39; wheat vs. maize: *p* = 0.97) (Figure 5A).

The response ratio of root fresh weight (RFW) for the same treatment comparison (Figure 5B). Similar to SFW, wheat (*p* = 0.0007, *k* = 4) and maize (*p* < 0.0001, *k* = 10) showed significant increases in root biomass under the combined treatment, while rice did not (*p* = 0.30, *k* = 2). Again, between‐group comparisons were not statistically significant (wheat vs. rice: *p* = 0.18; rice vs. maize: *p* = 0.15; wheat vs. maize: *p* = 0.98). In salinity‐stressed wheat and rice, limited studies were reported regarding the effects of seaweed extracts on their RDW (*k* = 4 and *k* = 2, respectively). As a result, further investigations are essential to elucidate the meticulous impacts of seaweed extracts on wheat and rice roots in a salinity environment.

### 3.5 Effects of Seaweed Extract on Cereal Growth Under Different Salinity Levels

**FIGURE 6:**
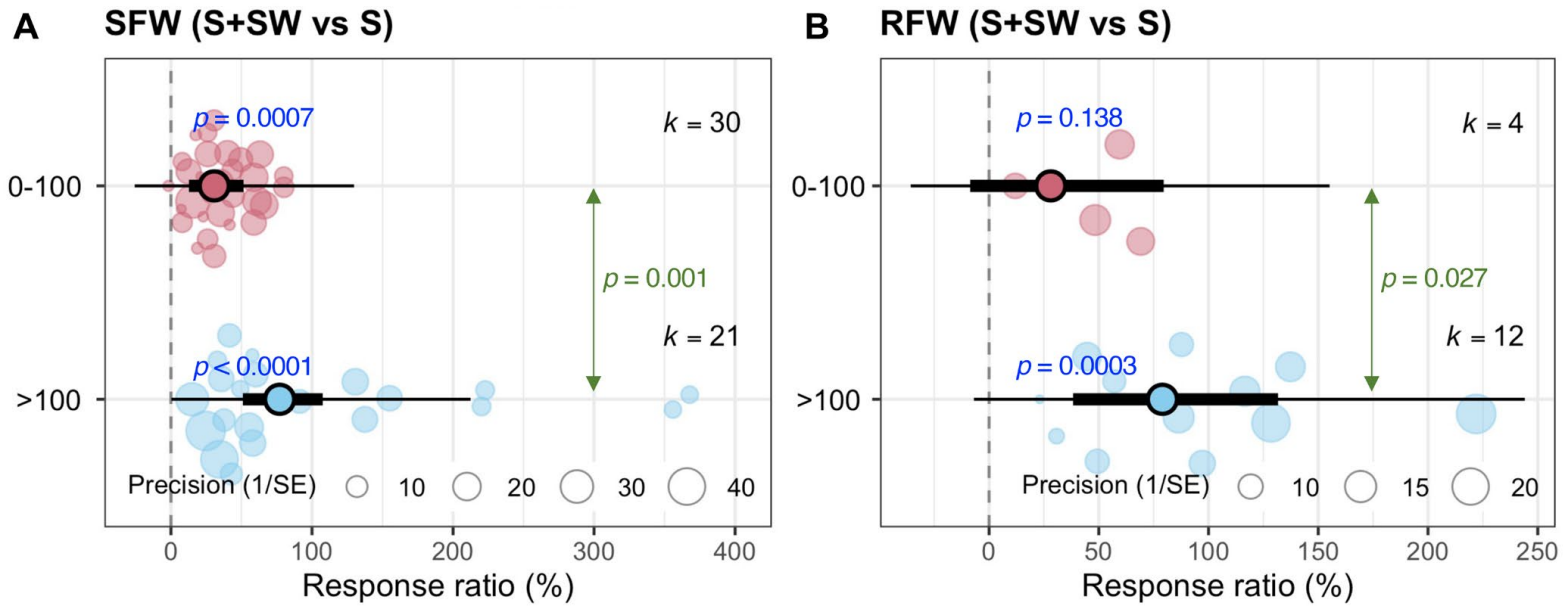
Effects of different salinity levels on seaweed extract‐medicated changes of shoot dry weight (SDW) and root dry weight (RDW) of varying cereal crops (wheat and maize). Plots show means, 95% confidence interval (CI, thick whisker), 95% prediction interval (PI, thin whisker), and individual effect sizes scaled by their precision (circles). *k* indicates the number of studies included in this study, and *p* < 0.05 indicates the significant effect of a particular treatment. The dashed vertical line shows the null response ratio (%). 0–100, 0–100 mM NaCl and > 100, > 100 mM NaCl. S (salinity) and S + SW (salinity + seaweed extract foliar spray) are different treatment combinations.

We compare the response ratios of SFW and RFW under the combined S + SW treatment relative to S alone, across two salinity level groups: 0–100 mM NaCl and > 100 mM NaCl. For SFW, the S + SW treatment led to a significant increase at both salinity levels, with a stronger effect observed under high salinity (> 100 mM; *p* < 0.0001, *k* = 21) compared to lower salinity (0–100 mM; *p* = 0.0007, *k* = 30). The difference between these two salinity groups was statistically significant (*p* = 0.001), indicating a greater efficacy of seaweed extracts for growth improvement under more severe salt stress (Figure 6A). For RFW, a significant increase in response to seaweed extract was observed only under high salinity conditions (*p* = 0.0003, *k* = 12), while the response under lower salinity was not significant (*p* = 0.138, *k* = 4). The between‐group comparison also revealed a significant difference (*p* = 0.027), suggesting that the positive effects of the S + SW treatment on root biomass are more pronounced under high salinity (Figure 6B).

### 3.6 Effects of Different Concentrations of Seaweed Extract on Cereal Growth

**FIGURE 7:**
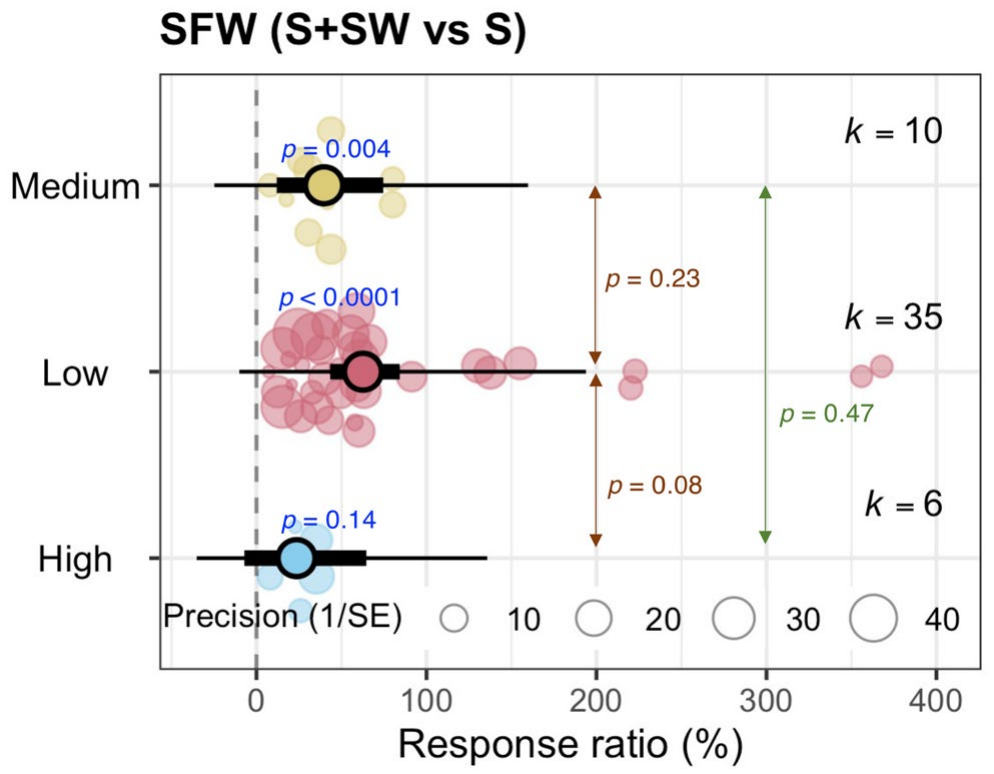
Effects of different concentrations of seaweed extract on shoot and root growth of varying cereal crops (wheat and maize) grown under salinity conditions. Orchard plot shows means, 95% confidence interval (CI, thick whisker), 95% prediction interval (PI, thin whisker), and individual effect sizes scaled by their precision (circles). *k* indicates the number of studies included in this study, and *p* < 0.05 indicates the significant effect of a particular treatment. The dashed vertical line shows the null response ratio (%). Low, 0.2%–10% SW extract; medium, 11%–25% SW extract; and high, > 25% SW extract. S (salinity) and S + SW (salinity + seaweed extract foliar spray) are different treatment combinations.

The effects of various concentrations (medium, low, and high) of seaweed extract on cereal crop growth were evaluated by sub‐group analysis (Figure 7). The low and medium concentrations of seaweed extract significantly increased (*p* < 0.0001 and *p* = 0.004, respectively) the SFW of cereals. However, a high level of seaweed extract did not substantially improve the SFW of cereals (*p* = 0.14) (Figure 7).

### 3.7 Robustness, Sensitivity, and Publication Bias of Estimated Effect Size

To assess publication bias, we performed Egger's test (Table S2). Among the treatment combinations, publication bias was found only in three treatment comparisons, S + SW vs. S (RFW, *p* = 0.0069), S vs. C (RDW, *p* = 0.0004), S + SW vs. S (RDW, *p* = 0.0045) (Table S2).

**FIGURE 8:**
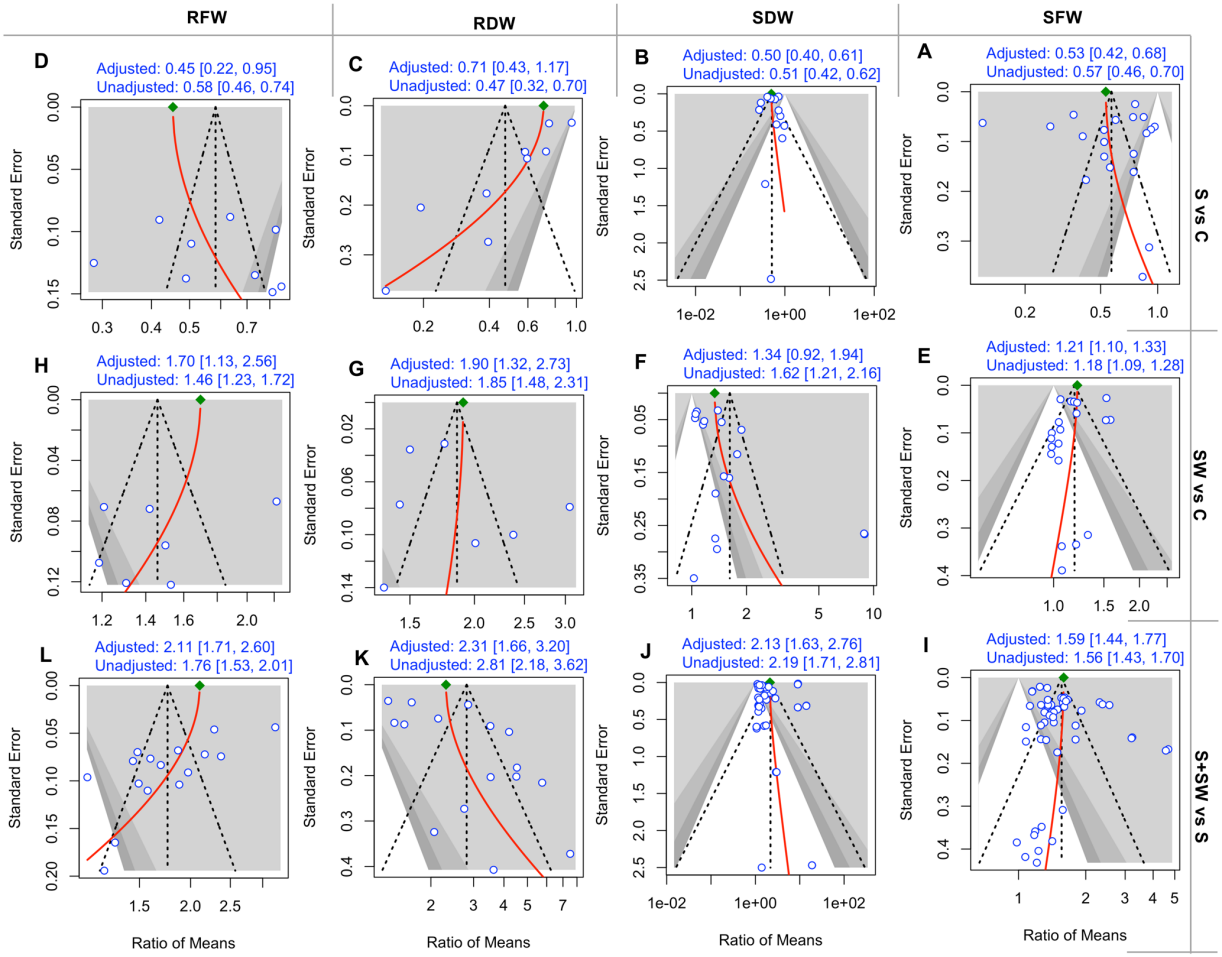
Contour‐enhanced meta‐analysis funnel plots from limit meta‐analysis assessing publication bias and adjusted effect size for three treatment comparisons: (a) salinity (S) vs. control (C), (b) seaweed extract (SW) vs. C, and (c) salinity + seaweed extract (S + SW) vs. salinity. Each plot displays the relationship between the ratio of means (effect size) and standard error, with adjusted effects accounting for potential publication bias.

We used another model, random effect model, for effect size estimation to check the robustness of our result and found that the unadjusted effect sizes are significant for all cases (Figure 8). Additionally, we conducted a limit meta‐analysis to further adjust for sensitivity and publication bias. Following a limit meta‐analysis adjustment, the effect sizes remained statistically significant in all cases, except for SW vs. C (SDW) (adjusted effect size: 1.34 and unadjusted effect size: 1.62) (Figure 8F).

## 4 Discussion

The meta‐analysis synthesizes data from diverse studies to draw more comprehensive and reliable conclusions. This study employed a systematic meta‐analysis approach to examine the effects of foliar spray of aqueous seaweed extracts on the growth of cereal crops. Our analysis revealed how different types and concentrations of seaweed extracts influence the growth of cereal crops under salinity stress, highlighting several research gaps that need to be addressed for further advancements in this field.

Salinity stress significantly reduces crop productivity by hampering morpho‐physiological processes (Negrão et al. 2017; Atta et al. 2023). Several studies have shown that salinity stress reduces biomass production in crops (Tahjib‐UI‐Arif et al. 2019; Dawood et al. 2021). In agreement with these studies, our meta‐analysis showed that exposure to salinity stress significantly reduced the SFW, SDW, RFW, and RDW of cereal crops (Figures 2A,D and 3A,D). The growth retardation occurred due to the induction of osmotic stress with over‐accumulation of Na^+^ and Cl^−^ ions in cells, resulting in ionic imbalance and toxicity (Han et al. 2015; Hussain et al. 2021). Under salinity stress, the plant metabolizes a finite amount of energy and resources instead of using them in the necessary processes for maintaining vegetative and generative growth (Munns and Gilliham 2015; Zörb et al. 2019). Our meta‐analysis demonstrated that the application of seaweed extracts significantly and positively affected SFW, SDW, RFW, and RDW under both non‐stress and salinity stress conditions (Figures 2B–F and 3B–F). Many studies have revealed that seaweed extracts function as potential biostimulants, containing phytohormones and polysaccharides that enhance crop growth by improving nutrient uptake, enhancing root growth, and increasing foliage levels (Tarakhovskaya et al. 2007; Abdel‐Latif et al. 2022). Overall, our analysis demonstrated that seaweed extracts enhance salinity tolerance in cereal crops, similar to alfalfa (El‐Sharkawy et al. 2017) and chickpea (Abdel Latef et al. 2017).

In our selected studies, seaweed extracts from three classes, *Phaeophyceae*, *Rhodophyta*, and *Chlorophyta*, were utilized to improve the salinity tolerance of cereal crops. Among these, seaweed extracts from *Chlorophyta*‐type seaweed were mainly used (Ibrahim et al. 2014; Latique et al. 2016, 2021; Attia et al. 2022; Chanthini et al. 2022). Our analysis revealed that water extracts from all three seaweed classes effectively alleviated salinity‐induced growth inhibition in cereal crops. However, the number of studies that used seaweed from *Rhodophyta*‐type seaweed is minimal to draw a conclusive conclusion (Figure 4). In the selected studies, water extracts from *Dictyota dichotoma* , *Chaetomorpha antennina* , *Hormophysa cuneiformis*, *Actinotrichia fragilis*, *Ascophyllum nodosum* , *Halimeda opuntia* , *Ecklonia maxima*, *Padina pavonica*, *Fucus spiralis* , *Ulva rigida* , and *Ulva lactuca* species were applied in rice, wheat, and maize for salinity tolerance improvement (Table S1).

Most of the selected studies investigated the effect of seaweed on the growth of wheat (Ibrahim et al. 2014; Latique et al. 2016, 2017, 2021; Abdel Latef et al. 2021), and only a few reported the impact on the growth of maize (Attia et al. 2022; Pienaar et al. 2025) and on rice (Shahzad et al. 2023). Based on the analysis, it is evident that seaweed extract application can improve the growth of wheat and maize but not rice (Figure 5). Throughout the selected studies, 34.2–400 mM NaCl equivalent salinity stress was applied (Table S1). Applying seaweed extracts mitigated the adverse effect of salinity on cereal growth both in low saline (0–100 mM NaCl) and high saline (100 mM NaCl) conditions, but the positive effect of seaweed extracts is more prominent in high saline conditions (Figure 6). The literature search showed that the researchers applied seaweed concentrations ranging from 0.2%–50% and extracted seaweed using water (Table S1). Within this range, ≤ 10% seaweed was mainly used, and our analysis showed that the low (0.2%–10%) and medium (10.1%–25%) levels of aqueous seaweed extract were effective in growth promotion under salinity stress in cereal crops (Figure 7). It should be noted that all syntheses were based on aqueous seaweed extracts applied as foliar sprays to cereal crops.

Positive or statistically significant findings often beckon greater attention, making them more likely to be published and ultimately included in meta‐analyses. This selection tendency can inadvertently inflate the perceived effectiveness of interventions, weaving a distorted tapestry of evidence. A comprehensive evaluation of meta‐analyses in plant ecology reveals that more than half of the meta‐analyses did not perform publication bias testing, and more than two‐thirds omitted sensitivity analyses (Koricheva and Gurevitch 2014). In contrast, our study embarked on a more rigorous path—intertwining both publication bias adjustments and sensitivity assessments into the analytical framework. Notably, the pooled effect sizes remained statistically robust even after navigating these methodological crucibles (Figure 8 and Table S2), underscoring the reliability and resilience of our findings. In addition, we recalculated the pooled effect size using a standard random‐effects model and observed a similar trend to that obtained with the multi‐level random‐effects meta‐analysis, further supporting the consistency of our findings (Figure 8). This lends further confidence that the treatment effects observed avoid potential biases, presenting a dependable narrative.

## 5 Limitations and Suggestions for Future Experiments

In the future, several key research areas should be thoroughly investigated to advance our understanding and application of seaweed extracts in agriculture. These areas include:

- The impacts of seaweed on the yield of cereal crops have yet to be studied extensively. Therefore, future studies should focus on examining how seaweed affects the yield of various cereal crops under salt stress.
- While a few studies have been conducted on maize and rice, research on oats, barley, and other cereal crops remains unexplored. Future research should investigate how rice, maize, barley, and oats respond to external applications of seaweed under salinity stress.
- Additional research is needed due to the lack of previous studies on the effect of *Rhodophyta*‐type seaweed extracts in cereal crops under salinity conditions.
- The effects of different seaweed extracts on root growth have not been studied extensively. Therefore, further research is needed to investigate the root growth characteristics of cereal crops in response to seaweed extract application.
- The conclusions of this meta‐analysis are based solely on the use of aqueous seaweed extracts. Future meta‐analyses should explore how different extraction methods impact cereal crop growth under salinity stress.
- Seaweed extracts can be applied using various methods; however, this analysis was based solely on studies involving foliar application. Further research is needed to evaluate how different application methods influence stress tolerance in cereal crops.

## 6 Conclusion

Overall, this meta‐analysis indicates that exogenous foliar application of aqueous seaweed extracts at ≤ 25% effectively mitigates the adverse effects of salinity on the growth of cereal crops.

## Conflicts of Interest

The authors declare no conflicts of interest.

## Supporting information


**Table S1:** List of selected studies used in this meta‐analysis.
**Table S2:** Heterogeneity and publication bias analysis.

## Data Availability

The data that support the findings of this study are openly available in ‘figshare’ at https://doi.org/10.6084/m9.figshare.30069079.v1.
