# Supplementary material for: Seaweed Extracts Improve Salinity Tolerance in Cereal Crops—A Meta‐Analysis
Source: Plant Environ Interact. 2025 Oct 28;6(5):e70094. doi: 10.1002/pei3.70094 (PMC12560104; doi:10.1002/pei3.70094)
Supplement: Supplementary file 1 — Table S1: List of selected studies used in this meta‐analysis. Table S2: Heterogeneity and publication bias analysis. [file PEI3-6-e70094-s001.docx]

**Supplementary files**

**Supplementary Table S1.** List of selected studies used in this meta-analysis.

| **Sl.**  **No.** | **Author** | **Publication year** | **Country of publication** | **Crop** | **Seaweed name** | **Seaweed type** | **Concentrations** | | **Ref. study** |
| --- | --- | --- | --- | --- | --- | --- | --- | --- | --- |
|  |  |  |  |  |  |  | **Seaweed (%)** | **Salinity (mM)** |  |
|  | Abdel Latef et al. | 2021 | Egypt | Wheat | *Hormophysa cuneiformis; Actinotrichia fragilis* | Phaeophytes (brown); Rhodophytes (red) | 1 | 100, 150 | Abdel Latef et al. 2021 |
|  | Attia et al. | 2022 | Egypt | Maize | *Halimeda opuntia; Padina pavonica* | Chlorophytes (green); Phaeophytes (brown) | 10 | 100, 200, 300, 400 | Attia et al. 2022 |
|  | Latique et al. | 2017 | Tunisia | Durum Wheat | *Fucus spiralis* | Phaeophytes (brown) | 5, 10, 25, 50 | 34.223, 68.446 | Latique et al. 2017 |
|  | Latique et al. | 2021 | Morocco | Wheat | *Ulva rigida* | Chlorophytes (green) | 12.5, 25, 50 | 34.22, 68.44 | Latique et al. 2021 |
|  | Ibrahim et al. | 2014 | Egypt | Wheat | *Ulva lactuca* | Chlorophytes (green) | 1, 5 | 50, 100, 150, 200, 250 | Ibrahim et al. 2014 |
|  | Latique et al. | 2016 | Morocco | Wheat | *Ulva rigida* | Chlorophytes (green) | 0.2, 0.5, 25, 50 | 34.223, 68.446 | Latique et al. 2016 |
|  | Pienaar et al. | 2025 | South Africa | Maize | *Ecklonia maxima* | Phaeophytes (brown) | 1 | 200, 300 | Pienaar et al. 2025 |
|  | Shahzad et al. | 2024 | Indonesia | Rice | *Ascophyllum nodosum* | Phaeophytes (brown) | 0.2, 0.4 | 200, 300 | Shahzad et al. 2024 |

**Supplementary Table S2.** Heterogeneity and and publication bias analysis.

| **Parameters** | **Treatment combination** | **Heterogenicity (Q) and p-value** | **Publication bias (Egger’s test)**  **and p-value** |
| --- | --- | --- | --- |
| SDW | S vs C | 170.9703, *p* < 0.0001 | 0.4345 *p* = 0.4417 |
| SDW | SW vs C | 234.2500, *p* < 0.0001 | 1.5645 *p* = 0.3654 |
| SDW | SW+S vs S | 16776.8815, *p* < 0.0001 | 0.2018 *p* = 0.6623 |
| SFW | S vs C | 1206.0593, *p* < 0.0001 | 0.0517 *p* = 0.9771 |
| SFW | SW vs C | 153.1582, *p* < 0.0001 | -0.5550 *p* = 0.4204 |
| SFW | SW+S vs S | 611.0447, *p* < 0.0001 | 0.3941 *p* = 0.5331 |
| RFW | S vs C | 77.5102, *p* < 0.0001 | 3.5344 *p* = 0.5626 |
| RFW | SW vs C | 50.0072, *p* < 0.0001 | -3.2306 *p* = 0.4496 |
| RFW | SW+S vs S | 244.0386, *p* < 0.0001 | -5.1678 *p* = 0.0069 |
| RDW | S vs C | 142.1703, *p* < 0.0001 | -5.4169 *p* = 0.0004 |
| RDW | SW vs C | 89.5828, *p* < 0.0001 | -9.7473 *p* = 0.0557 |
| RDW | SW+S vs S | 413.4530, *p* < 0.0001 | 3.3174 *p* = 0.0045 |

**References**

Abdel Latef, A. A. H., Zaid, A., & Alwaleed, E. A. (2021). Influences of priming on selected physiological attributes and protein pattern responses of salinized wheat with extracts of *Hormophysa cuneiformis* and *Actinotrichia fragilis*. *Agronomy*, *11*(3), 545.

Attia, E. Z., Youssef, N. H., Saber, H., Rushdi, M. I., Abdel-Rahman, I. A., Darwish, A. G., & Abdelmohsen, U. R. (2022). *Halimeda opuntia* and *Padina pavonica* extracts improve growth and metabolic activities in maize under soil-saline conditions. *Journal of Applied Phycology*, *34*(6), 3189-3203.

Latique, S., Mohamed Aymen, E., Halima, C., Chérif, H., & Mimoun, E. K. (2017). Alleviation of salt stress in durum wheat (*Triticum durum* L.) seedlings through the application of liquid seaweed extracts of Fucus spiralis. *Communications in Soil Science and Plant Analysis*, *48*(21), 2582-2593.

Latique, S., Mrid, R. B., Kabach, I., Kchikich, A., Sammama, H., Yasri, A., ... & Selmaoui, K. (2021). Foliar application of *Ulva rigida* water extracts improves salinity tolerance in wheat (*Triticum durum* L.). *Agronomy*, *11*(2), 265.

Ibrahim, W. M., Ali, R. M., Hemida, K. A., & Sayed, M. A. (2014). Role of *Ulva lactuca* extract in alleviation of salinity stress on wheat seedlings. *The Scientific world journal*, *2014*.

Latique, S., Chernane, H., Mansori, M., & El Kaoua, M. (2016). Biochemical modification and changes in antioxidant enzymes in *Triticum durum* L. by seaweed liquid extract of Ulva rigida macroalgae under salt stress condition. *Adv. Environ. Res*, *50*, 35-54.

Pienaar, B. C., Majeke, B. M., Wittenberg, M. F., Adetunji, A. E., Nephali, L., Tugizimana, F., & Rafudeen, M. S. (2025). Mitigating salt stress in maize using *Ecklonia maxima* seaweed extracts. *Plant Stress*, 16, 100828.

Shahzad, R., Harlina, P. W., Gallego, P. P., Flexas, J., Ewas, M., Leiwen, X., & Karuniawan, A. (2023). The seaweed *Ascophyllum nodosum*-based biostimulant enhances salt stress tolerance in rice (*Oryza sativa* L.) by remodeling physiological, biochemical, and metabolic responses. *Journal of Plant Interactions*, 18(1), 2266514.
